# Improving prevalence estimation through data fusion: methods and validation

**DOI:** 10.1186/s12911-015-0169-z

**Published:** 2015-06-24

**Authors:** Tomàs Aluja-Banet, Josep Daunis-i-Estadella, Núria Brunsó, Anna Mompart-Penina

**Affiliations:** 1 Universitat Politècnica de Catalunya, Campus Nord, C5-204, Barcelona, E-08034 Spain; 2 Universitat de Girona, Campus de Montilivi, Edifici Politècnica 4, Girona, E-17071 Spain; 3 Institut d’Estadística de Catalunya, Via Laietana 58, Barcelona, E-08003 Spain; 4 Departament de Salut, Generalitat de Catalunya, Travessera de les Corts, 131-159, Barcelona, E-08028 Spain

**Keywords:** Population surveys, Prevalences, Diabetes, Cardio vascular diseases, Multiple imputation, Sequential regression

## Abstract

**Background:**

Estimation of health prevalences is usually performed with a single survey. Some attempts have been made to integrate more than one source of data. We propose here to validate this approach through data fusion. Data Fusion is the process of integrating two sources of data into one combined file. It allows us to take even greater advantage of existing information collected in databases. Here, we use data fusion to improve the estimation of health prevalences for two primary health factors: cardiovascular diseases and diabetes.

**Methods:**

We use a real data fusion operation on population health, where the imputation of basic health risk factors is used to enrich a large-scale survey on self-reported health status. We propose choosing the imputation methodology for this problem through a suite of validation statistics that assess the quality of the fused data. The compared imputation techniques have been chosen from among the main imputation methodologies: k-nearest neighbor, probabilistic modeling and regression. We use the 2006 Health Survey of Catalonia, which provides a complete report of the perceived health status. In order to deal with the uncertainty problem, we compare these methodologies under the single and multiple imputation frames.

**Results:**

A suite of validation statistics allows us to discern the strengths and weaknesses of studied imputation methods. Multiple outperforms single imputation by providing better and much more stable estimates, according to the computed validation statistics. The summarized results indicate that the probabilistic methods preserve the multivariate structure better; sequential regression methods deliver greater accuracy of imputed data; and nearest neighbor methods end up with a more realistic distribution of imputed data.

**Conclusions:**

Data fusion allows us to integrate two sources of information in order to take grater advantage of the available data. Multiple imputed sequential regression models have the advantage of grater interpretability and can be used for health policy. Under certain conditions, more accurate estimates of the prevalences can be obtained using fused data (the original data plus the imputed data) than just by using only the observed data.

## Background

### Overview of the problem

Large-scale surveys based on interviews are used as a tool to assess the health of the population. These surveys provide large representative samples of the population of interest. Obtained data are based on questions and self-reported answers. This kind of data could lead to inaccurate and biased estimates of health condition and risk factor measures: respondents frequently misreport height and weight, or the absence of local services can lead to them reporting that they do not use these services [1]. On the other hand, more accurate clinical data, are obtained in small surveys, due to their greater cost.

This paper describes research on methods that combine clinical values with a large-scale sample of self-reported questions. Data Fusion techniques are used as a tool for integrating information from different sources in order to improve the estimation of the prevalences. Data fusion is a technological operation undertaken for specific operational purposes, with the aim of gaining more information about specific queries of interest from the existing data. The imputations are created using models that have been fitted from a survey that contains both self-reported and clinical data.

Large-scale public surveys can be enriched by data fusion from a small-scale survey with health risk indicators. We present an illustrative study which employs the 2006 Health Survey of Catalonia (ESCA) for the large-scale survey, and a subsample from this survey for the small-scale survey. The former is a complete report of perceived health status, whereas the latter is a collection of clinical data obtained from a health exam data EXCA. The health conditions considered are cardiovascular diseases (CVD) and diabetes.

The question to answer is whether fused data files deliver better estimates of prevalences than only the observed data from a small-scale survey [2].

### Theoretical aspects of data fusion

Data fusion, also known as statistical matching, is a technological operation whose aim is to integrate the information of two independent data sources. Technically, it involves the imputation of a complete block of missing variables. Its main applications are in media surveys, where they are used to integrate consumption data with audience data, and in National Statistical Institutes, where reducing the increasing burden generated by official statistics is a difficult problem to overcome.

**Fig. 1:**
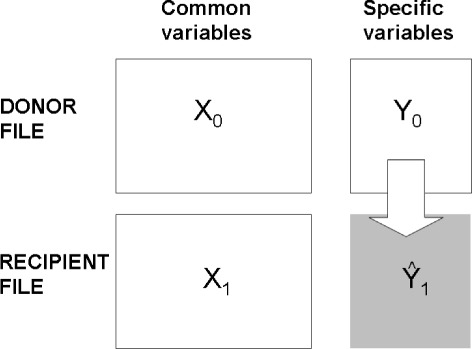
Unilateral fusion diagram. The unilateral data fusion transfers the specific variables of the *donor file* (with *X* and *Y* variables) to the *recipient file* (with just *X* variables) at individual level

Here, we address the problem in its simplest case, called unilateral fusion (Fig. 1), in which there are two files: one with *X* and *Y* variables (*donor file D:X*_0_;*Y*_0_), and the other with only *X* variables (*recipient file R:X*_1_). The *X* variables are currently called *common, link, hinge or bridge variables*, while the *Y* variables are the *specific, imputing or fusing variables*. The objective of the data fusion is to transfer the specific variables of the *donor D*file to the *recipient R*file at the individual level. Nevertheless, as Saporta states, imputed data is not “real” data but estimates. Hence, one has to be very careful when using such data which can only be used at aggregated level [3].

Let *f*(*X*,*Y*) be the joint (unknown) density function. Let *n*_0_ and *n*_1_ be the sizes of the donor and recipient files respectively. The goal is to complete the recipient file $(X_{1},\hat {Y}_{1})$ in such a way that it can a be an instance of *f*(*X*,*Y*).

In data fusion, we do not need to assume that both files are samples drawn independently from the same parent population. In fact, recipient files may not be a representative sample at all [4]. Our aim is to complete the information of the recipient file in order to take advantage of the existing relationship between the donor and recipient data. This allow us to obtain more accurate solutions for the research undertaken here. However, in order to perform a valid transfer of information, we need the donor file, *D*, to be a representative set of the parent population, from which inferences can be made.

A crucial first step is to assess the validity of the imputed data. Thus, we classically employ the conditional independence assumption [5], which implies that conditional to *X*, there are no more variables related to *Y*, in other words, given any set of variables *Z*, we have *f*(*Y*,*Z*|*X*)=*f*(*Y*|*X*)·*f*(*Z*|*X*). We prefer to reformulate the same principle by saying that the *X* variables account for all significant variability of the *Y* variables, given the imputation model. That is, *Y*=*i*(*X*)+*ε*, where *i*(*X*) stands for the imputation model and *ε* just conveys random fluctuations. We call this assumption the “predictive relevance” of the common variables (with respect to the specific ones).

The goal of data fusion is usually to simulate real data, which implies reproducing the conditional distribution of donors among the recipients

$$f(\hat{Y}_{1}|X_{1}) = f(Y_{0}|X_{0}). $$

However, in some cases the practitioner may be interested not in simulating real data, but in minimizing the prediction error $E[Y_{1}-\hat {Y}_{1}]^{2}$. This implies performing a deterministic imputation $\hat {Y} =i(X)$. As proven by Aluja et al. [6], both objectives cannot be jointly optimized.

Different imputation methods are possible when dealing with a specific problem. In order to help health policy researchers choose the most appropriate imputation method for a specific problem, we propose using a suite of validation statistics that measure some facets of the goodness of the fused data.

### Contents of the paper

The paper is organized as follows. In the next section, we present the data and provide the definition of the conditions considered. Section ‘Results’ describes the key points of data fusion and imputation methods. In Section ‘Discussion’, we deal with the problem of assessing the goodness of fit of fused data by means of a suite of validation statistics, and we consider the uncertainty problem of fused data. In section ‘Conclusions’, we compare six fusion models, (the most commonly used in research) by employing public health survey data to apply the aforementioned validation tools and the methodology for coping with the uncertainty problem. We use the selected imputation model to perform data fusion on the clinical data, and then we compare the prevalences of CVD and diabetes that are obtained from the fused data and the small-scale survey of clinical data. Finally, we close with some practical conclusions.

## Methods

### Data collection: the ESCA and the EXCA surveys

The 2006 Health Survey of Catalonia [7] (ESCA from now) is a multipurpose health survey comprising a representative sample of individuals, from which a complete report of their perceived health status is taken. It surveys roughly 19400 people each year, in 37 primary sampling units. The sample size is calculated by allocating a minimum number of respondents to each primary sampling unit in order to achieve a margin of error of around 5 *%*, enough to get consistent and statistically significant indicators at these sampling unit levels. The ESCA contains questions on socio-demographic characteristics, health status, activity limitations, use of health services, and health care, all of which are asked to every individual in the survey. The overall objective of the ESCA is to ascertain health status, lifestyle and the use of health services. The overall purpose of ascertaining these parameters is to identify health and services needs, establish different population profiles, evaluate goals for reducing health risk, and evaluate the effectiveness of health interventions. Within that survey, a subsample of around 1900 individuals (Health Exam data [7], EXCA from now) is taken to obtain measures of the basic risk factors. The physical examinations consist of medical examinations and laboratory tests: systolic blood pressure, diastolic blood pressure, glycemia, cholesterol and body mass index.

The present work has been carried out under the framework of a Research Agreement between the IDESCAT (Catalan Institute of Statistics) and the UPC (Universitat Politècnica de Catalunya − Barcelona Tech). To this end, the main objective was the integration of the Health Survey 2006 with the Examination Survey 2006. The IDESCAT granted the access to the data and the field expertise through the Health Department of the Catalan Government.

People under 18 were excluded from the research data set as well as people with self-reported, laboratory or physical examinations outside the normal intervals. Thus, the final sample size of the used data consisted of 11614 people for the ESCA and, among them, 1508 pertained to the EXCA.

The common variables that were used from the two surveys are listed in Table 1.

**Table 1:** Common variables used

Variable	Modalities	Variable	Modalities
Gender	1=’Female’	Diabetes	1=’No Diab’
	2=’Male’		2=’Diab without treat’
Age	1=’18:29 years’		3=’Diab with treat’
	2=’30:44 years’	Cholesterol	1=’No high Chol’
	3=’45:59 years’		2=’Chol without treat’
	4=’60:74 years’		3=’Chol with treat’
	5=’75 years & over’	Blood pressure control	1=’No BPC’
Age	Continuous		2=’Yes BPC’
Self-reported height	Continuous	Cholesterol control	1=’No CC’
Self-reported weight	Continuous		2=’Yes CC’
Blood pressure	1=’No high BP’	Physical activity	1=’Sedentary’
	2=’BP without treat’		2=’Light’
	3=’BP with treat’		3=’Moderate’
Smoking	1=’Non smoker’		4=’Vigorous’
	2=’Ex-smoker’	Alcohol intake	1=’Non drinker’
	3=’Occasional smoker’		2=’Moderate drinker’
	4=’Regular smoker’		3=’Risk drinker’

The subjects’ birth date was replaced by age group.The Blood Pressure variable (BP) is divided into three categories: (1) BP declared not high, (2) BP declared high but without taking any medicine, and (3) BP declared high and taking medicine for it. The same coding is applied to diabetes and cholesterol. Occasional smoker refers to smoking less than one cigarette per day. Light physical activity means standing during the greater part of the work day or doing physical activity or sport at least one day a week (for at least 20 minutes); Moderate physical activity refers to activity that usually does not require considerable physical effort, but the respondent walks frequently or does physical activity or sport more than one day a week; Vigorous physical activity indicates that the usual activity requires intensive physical effort. Alcohol intake classifies respondents according to their weekly intake of pure alcohol, where the moderate drinker category corresponds to less than 280 gr. per week for men and 170 gr. per week for women. Risk drinkers are above these quantities.

### Risk factors and definitions of conditions

The purpose here is to study the prevalences of the two considered conditions, cardiovascular diseases and diabetes in the 2006 Catalan population. Their risk factors are in Table 2. The clinical classification of hyperglycemia was based on fasting plasma glucose levels greater than or equal to 126 mg/dL, or on taking medication for diabetes. A person’s hypertension classification was based on systolic blood pressure greater than 140 mmHg or diastolic blood pressure greater than 90 mmHg (135 mmHg and 85 mmHg respectively for diabetic persons), or on taking medication to control blood pressure. Cholesterolemia was based on having more than 250 mg of cholesterol per deciliter of blood (mg/dL). Obesity was defined as having a body mass index (BMI) greater than 25, where BMI = (weight in kg)/(height in meters) ^2^. In calculating BMI, either self-reported height and weight or measured (clinical) height and weight could be used. Abdominal obesity was defined as having an abdominal perimeter greater than 102 cm for men or greater than 88 cm for women.

**Table 2:** Risk factors considered

Risk factors for cardiovascular diseases	Risk factors for diabetes
Gender	Gender
Age	Age
BMI	BMI
Cholesterolemia	Cholesterolemia
Physical activity	Physical activity
Abdominal obesity	Abdominal obesity
High blood pressure	High blood pressure
Educational Attainment	Educational Attainment
Smoking	
Hyperglycemia	

Self-reported conditions were available for persons in both the ESCA and the EXCA, whereas clinical conditions were available only for persons in the EXCA subsample.

### Imputation methods

There are three main basic approaches to data fusion. The first consists of embedding the common and specific variables into a *parametric* multivariate distribution *f*(*X*,*Y*|*θ*) assuming donors and receptors independently and randomly drawing from this distribution. This distribution can be factored into *f*(*X*,*Y*|*θ*)=*f*(*Y*|*X*,*θ*_*Y*|*X*_)*f*(*X*,*θ*_*X*_). Hence, it is possible to estimate its parameters *θ*_*X*_ and *θ*_*Y*|*X*_ from the available information in files *D* and *R*, respectively, and then use them to impute the missing block of data [8]. The second approach consists of directly *modelling*the relationship between the *Y* and *X* variables in the donor file D by means of a regression function, *E*(*Y*|*X*)=*r*(*X*)+*ε*, and then applying this model to the recipient file R (*explicit modelling*). The last approach consists of finding, for each individual of the recipient file, one or more donor individuals that are as similar as possible, and to then transfer in some way, the values of the *Y* variables to the *recipient*individual (*implicit modelling*). This method is known as *hot deck*, a term borrowed from data editing in data bases.

The *parametric* imputation is bound to the missing data problem. It assumes a common distribution *f*(*X*,*Y*|*θ*) for the donor and recipient files. Then it maximizes the observed likelihood $f(Y|X, \hat {\theta }_{Y|X})$. It usually uses the EM algorithm [9] which allows us to obtain an imputation by iterative optimization of the complete likelihood. Another useful parametric imputation is the data augmentation (DA) algorithm [10]. The DA algorithm is intended to simulate instances of the distributions by allowing for the variability of the parameters themselves. The DA algorithm can be easily performed for a multinormal distribution in the case of specific continuous variables or for a multinomial distribution in the case of categorical variables [11, 12]. Notice that DA gives us a distribution of imputed values (i.e. the possibility of obtaining as many stochastic imputations as we want), whereas EM provides only one single deterministic imputation.

*Explicit modelling* can be performed by any multivariate regression technique (ordinary least squares, sequential regressions, partial least squares, decisions trees, etc.). It skips over the problem of formulating a parametric model, although it can easily be shown that in the case of multivariate normality both approaches are equivalent [8]. In this case, the recipient file is not a necessary representative of the population. It is worth to note the approach based in resampling decision trees under the statistical learning theory, named BINPI-Fusion, where the imputation consist in minimizing the prediction error of the imputed values [13].

*Hot deck* is the simplest and most flexible method, because it requires no assumptions about the probabilistic distribution or about the formal relationship between the specific and common variables. It is a data-based method and, in this sense, it is distribution free. It can be a *random hot deck* when the donor (or donors) within a specific group are selected at random through some characteristics they share with the recipient; or it can be a *distance hot deck* (better known as the *k*nn method), where the donor and recipients are placed in a common subspace defined by the common variables. Then, for each recipient, the *k*-nearest donor neighbors are found and listed [8]. Or a mixed methodology of both approaches can be applied: a method based on distance selection within a class of individuals [6]. In this method we do not need both files to be representatives of the population, we only need all groups to be present in the donor file and for them to cover the whole range of the population. Finally, the assignment is made based on the list of neighbors. Clearly, the hot deck methodology implies performing random draws from the empirical conditional distribution $\hat {f}(Y|X,\theta _{Y|X})$.

### Validity and uncertainty of imputed data

Once an imputation has been performed, we need to assess the validity of the operation. We will say that the data fusion is valid if the fused data set $(X_{1},\hat {Y}_{1})$ is an instance of the distribution function *f*(*X*,*Y*). In general, the distribution function *f* is unknown; thus we are compelled to compare the empirical distribution functions $\hat {f}(X_{0},\hat {Y}_{0})$ with $\hat {f}(X_{0},Y_{0})$.^1^

We call the discrepancy between both distributions *matching noise*. Following D’Orazio et al. [8], Conti et al. [14] and Paass [15], the matching noise depends on the correctness of the imputation function *i*(*X*) in approximating instances with the true conditional distribution *f*(*Y*|*X*).

### Validation tools

To calibrate the quality of the fused data that is produced, we need to gauge it as an actual instance of *f*(*X*,*Y*). Several suites of statistics have been proposed for this purpose [4, 6, 13, 16, 17], and there are therefore many ways to validate the imputed data. However, the best ones are probably those related to the final utility of data. We have defined a series of statistics [18] in order to perform a complete validation of the imputed data, and we have further grouped these statistics according to different criteria that can be achieved from a statistically neutral point of view:

- A. Preservation of global marginal statistics
  - 1. Comparison of marginal statistics [ASLm, ASLs] (means and standard deviations)
- B. Preservation of multivariate data distribution
  - 2. Comparison of the correlation between specific variables [ACDi]
  - 3. Comparison of the correlation between specific and common variables [ACDe]
  - 4. Comparison of the multivariate pattern of variability [WC]
- C. Preservation of imputed distributions
  - 5. Comparison of the distribution of observed and imputed variables [ASD]
- D. Accuracy of imputation
  - 6. Calculation of prediction error [TAU]
  - 7. Evaluation of the randomness of residuals [RndRes]

To put it simply, these statistics merely consist of running the univariate comparisons tests of the fused data set $(X_{0},\hat Y_{0})$ with the observed data (*X*_0_,*Y*_0_), and averaging the result. The reason for this is that the multivariate parametric hypothesis becomes unrealistic when the problem grows in complexity. A more detailed description about these statistics is provided in the aforementioned reference. These statistics provide a very complete validation set of the imputed data, which assures the coherence of the imputed values regarding the collected data from donors. However, as [12] pointed out, the correctness of the imputation model does not precludes correctness of the foreseen statistical analysis of fused data. For that reason, we have compared the relative change in the variances of CVD and diabetes prevalences in order to assess the value of the fusing operation in this case.

### The uncertainty problem

However, whatever the imputation method chosen, imputed data is not like observed data because it has inherent uncertainty. Imputed values $\hat {Y}_{1}$ are estimates; thus, to be realistic, we need to take into account the variability of the imputed data when analyzing it. This variability comes from the random fluctuation of the distribution *f*(*Y*|*X*,*θ*_*Y*|*X*_) and also from the fact that model parameters *θ*_*Y*|*X*_ are unknown. Hence, we have to work with estimates that, as a consequence, also convey random fluctuation.

Multiple Imputation (MI) is the classical way to cope with this problem [11, 16, 19–21]. It consists of repeating the single imputation procedure several times, from the predictive distribution of *f*(*Y*|*X*,*θ*_*Y*|*X*_), doing so under the realistic conditions of parameters *θ*_*Y*|*X*_. Then, we just concatenate the several single imputation files into one file. Following this, we apply the Multiple Imputation method to our Data Fusion problem and, according to Rubin [11], we can compute the variability of our estimates. This is composed of two terms: the within variability, which is the average sampling variability and is related to the size of the recipient sample; and the between variability, which is the variability related to the different multiple imputations.

## Results

Our purpose here is twofold. First, we want to select a particular model for imputation. Then, we will take CVD and diabetes in the fused data file and compare their precision with that of the EXCA file.

### Application to health survey data: the process

For our imputation models, we have selected a parametric imputation method (using the Data Augmentation algorithm (DA)), a sequential regression of fusing variables (SQ-reg), and a stochastic hot deck imputation, which is classically obtained through the nearest neighbor algorithm (1nn). All these methods are compared using single imputation and multiple imputation in order to assess the gain induced by the multiple imputation. We thus compare the following imputation methods given in Table 3.

**Table 3:** Imputation methods compared

Simple Imputation	Multiple Imputation (MI)
1nn	MI *k*nn (MI-*k*nn)
Sequential regressions (SQ-reg)	MI Sequential regressions (MI-SQ-reg)
Data Augmentation (DA)	MI Data Augmentation (MI-DA)

We emphasize here that we are interested mainly in the preservation of the multivariate structure of the imputed data, in order to simulate instances of real data. This implies using a stochastic imputation. In all of the three considered scenarios, we have performed stochastic imputation by random draw of suitable conditional distributions. Thus, we do not consider the imputation through conditional means, such as EM algorithm, deterministic regression or 10nn methods, even though that would improve the accuracy of the fused data. These methods would be beyond the scope of the this paper.

To have an insightful comparison, we have worked with bootstrap resampling, in order to assess the aforementioned validation statistics’ variability among the different imputation methodologies. Since the only available complete information is in the EXCA subsample, validation has been performed by comparing the imputed values of these individuals with the actual values (validation performed on donors only).

The process was as follows:

Iterate 400 times

(i) Extract bootstrap samples from the donor and recipient files.
(ii) Perform a single imputation with DA, SQ-reg and 1nn.
(iii) Perform a multiple imputation with MI-DA, MI-SQ-reg and MI-knn, 20 times.
(iv) Compute the suite of validation statistics ASLm, ASLs, ACDi, ACDe, WC, ASD, TAU, and RndRes.

### Main results

**Fig. 2:**
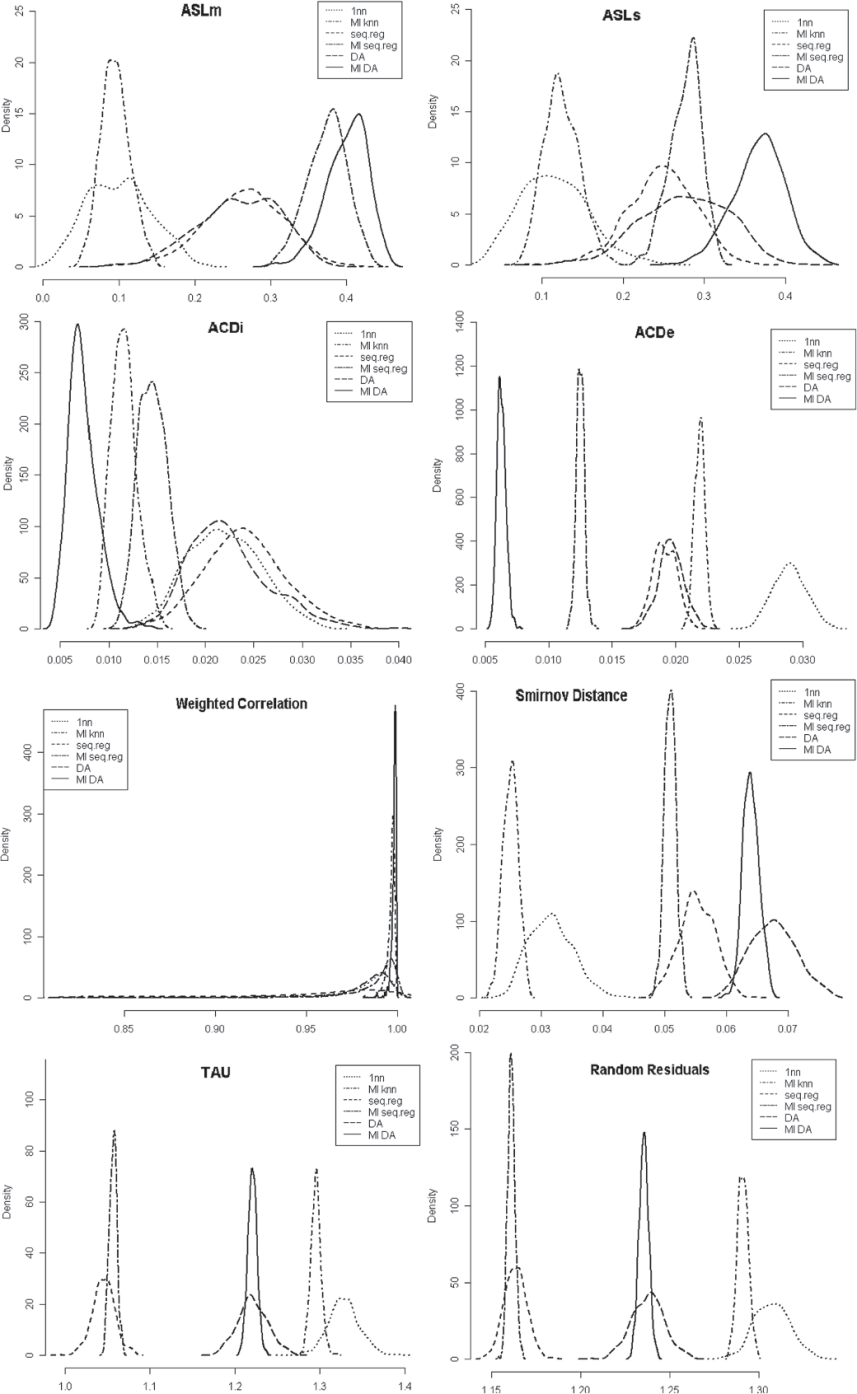
Densities of validation statistics obtained in 400 bootstrap samples. Legend: solid line=MI-DA, long-dashed line=DA, long-dashed dashed line=MI-SQ reg, dashed line=SQ reg, dotdashed line=MI-*k*nn, dotted line= 1nn method

For the selected imputation methods, we obtained the density function of the 8 validation statistics in the 400 bootstrap resamples (Fig. 2). Comparison of these densities reveals the performance of each method under a given statistic. Both the average value and the variability (as manifested in the shape of the density function) provide goodness in regard to the different validation criteria.

Note that the results of multiple imputation distributions are clear: the multiple imputation not only improves upon single imputation, by providing better validation statistics, but the validation statistics are much more stable than those from a single imputation [18].

Comparing the three imputation methodologies, we can see that DA, which is based on the probabilistic distribution of data, better preserves the multivariate structure of the imputed data. Sequential regression excels in predicting more accurate values, that are closer to the true ones. And the nearest neighbor methodology delivers real values that are distributed similarly to the original ones.

Going further, we can see that nearest neighbor provides more biased global statistics, the worst accuracy and randomness of residuals. But it gives us the best matching (lowest ASD). Thus, the marginal distribution of imputed values follows the true ones more closely.

The sequential regression approach excels in accuracy (TAU) and the randomness of residuals; hence, they impute values that are closer to the true ones. However, the multivariate distribution of imputed values is the farthest from to the original ones (lowest WC).

The Data Augmentation algorithm provides the best results with respect to the precision of the global statistics (ASLm, ASLs), the pairwise correlations (ACDi, ACDe) and the similarity between both multivariate distributions (WC). But it fails in the matching, delivering the farthest univariate empirical distributions (lowest ASD).

However, the MI-Seq-Reg presents the advantage of model interpretability, which in itself is useful for health policy. Thus, MI-Seq-Reg was the method selected for imputation in this case.

### Sequential regression models

Sequential regression modeling is a technique that estimates a set of values for each variable. It uses a regression model whose predictors include estimated values for other variables through other regression models. The goal here is to obtain multiple draws from the predictive distribution for each person in the ESCA.

The imputation in the ESCA file of CVD and diabetes conditions was conducted in two phases:

- First, we have performed 20 stochastic imputations (multiple imputation) of the risk factors by means of sequential regressions.
- Second, we have obtained the risk prediction of CVD and diabetes for each imputed individual.

The imputation models do not preclude the models for analysis. However, they need to be coherent with the foreseen analysis of the fused data. That is, if we want to breakdown the results by a factor, this factor must be included as a bridge variable in the imputation model (i.e., as a predictor, if we use a regression imputation model).

**Fig. 3:**
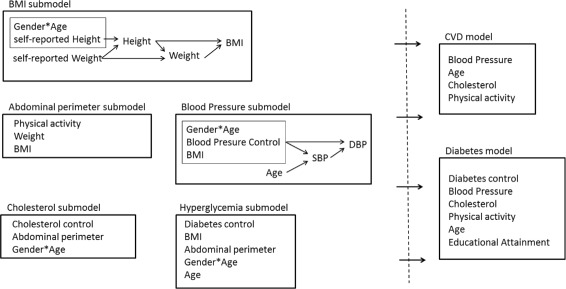
Sequential regression model scheme. Scheme of sequential regression models obtained by following two rules for including predictors: epidemiological/medical sense and statistical significance

In Fig. 3, we display the scheme of obtained sequential regression models. In any case, the sequential regression models have been defined according to two rules: (1) they must have epidemiological and medical sense, and (2) only statistically significant predictors can be included. In all cases, we performed a stochastic imputation to assure the simulation of real data instances for ESCA individuals.

The sequential regression model starts with the prediction of height. The height model could be constructed by fitting a regression model to the EXCA data, with height as the outcome variable. Because the following are the only significant predictors, we can use self-reported height, self-reported weight, and the interactions between gender and categorized age. Then, for each person in the ESCA, that is not present in the EXCA survey, the fitted regression allowed us to draw values of height for 10106 individuals.

Once we have height values, we use these values and the self-reported weight to obtain a fitting model to predict weight values for the ESCA data. Finally, with weight and height values, BMI is obtained as usual (Fig. 3, BMI submodel).

All the other submodels (abdominal perimeter, blood pressure, cholesterol and hyperglycemia) are constructed in the same way.

For individuals in the ESCA, cardiovascular disease (CVD) and diabetes status was multiply imputed using a logistic regression model. Their predictors are given in their respective models (Fig. 3, right).

### Imputed prevalence rates

With imputed data obtained through MI sequential regression, we estimate the prevalence rates for cardiovascular disease and diabetes, and their standard deviation (SD), following Rubin’s approach from Section ‘The uncertainty problem’.

Table 4 contains estimated global prevalence rates of CVD and diabetes, which are also broken down by sex, age, and physical activity. We compute both prevalences and their standard deviations from the EXCA and from the data fusion of EXCA plus ESCA (that is, the EXCA individuals plus the ESCA individuals for whom we have multiple imputations of their risk factors and clinical measures). Dividing the standard deviation of the EXCA prevalence by the standard deviation of the EXCA plus ESCA prevalence, we obtain the SD ratio. That is, to assess the value of the fusing operation, we compare the single source case (only EXCA data) with the completed case (EXCA plus fused ESCA data), specifically by looking at the relative change in the standard deviation of CVD and diabetes prevalences.

**Table 4:** Prevalence rates of cardiovascular disease and diabetes

		Cardiovascular disease			Diabetes		
Categories		Exca	Exca+Esca	Ratio of SD	Exca	Exca+Esca	Ratio of SD
Global		0.1054	0.1027	1.67	0.0643	0.0655	1.42
Gender	Male	0.1133	0.0980	1.71	0.0600	0.0651	1.48
	Female	0.0976	0.1071	1.56	0.0686	0.0658	1.40
Age	30-44	0.0191	0.0210	1.27	0.0122	0.0178	1.14
	45-59	0.0751	0.0620	1.66	0.0419	0.0403	1.44
	60-74	0.1935	0.1643	1.68	0.1310	0.1133	1.59
	75 & more	0.3427	0.3185	2.14	0.1888	0.1754	1.56
Physical	Vigorous	0.0658	0.0485	1.56	0.0526	0.0332	1.61
activity	Moderate	0.0759	0.0750	1.69	0.0422	0.0483	1.36
	Sedentary	0.2000	0.1905	1.66	0.1315	0.1196	1.67

We can see that the estimates of CVD are similar (0.1054 vs. 0.1027), but the EXCA plus ESCA is more precise (ratio of SD =1.67). Likewise with diabetes: 0.0643 vs. 0.0655 with ratio of SD =1.42.

For every combination of condition and subgroup in Table 4, the estimated prevalence from the multiply imputed clinical data (EXCA plus ESCA) is equivalent to that from the observed clinical data (EXCA). Note, however, that the ratios of estimated standard errors of estimated prevalence rates are greater than one. That is, the prevalence rates estimated in the fused file are more precise than those estimated with only the clinical data. Equivalent results were obtained by Schenker et al. [2].

## Discussion

The purpose of health surveys is to capture the health status of a population and their associated risk factors in a given period for the purpose of taking intervention measures that will improve it. Classically, this can be achieved with a large single survey. However, from a practical perspective, obtaining representative clinical data in a large sample is too expensive; in such a situation, making use of the integration techniques of different data sources can be useful and rewarding, provided that the process meets some quality requirements.

In our case, clinical data can only be available for a subsample (EXCA), due to its cost. Thus, it is worth matching this information statistically with the large and light health survey (ESCA), with which a suite of validations tools, can obtain better estimates of the CVD and diabetes prevalences for the Catalan population.

The results obtained from the ESCA/EXCA imputation allow us to infer the advantages and disadvantages of the different imputation models used.

Summarizing the obtained results, probabilistic imputation models better preserve the multivariate distribution of the data and marginal statistics, whereas regression models provides greater accuracy of imputed values; and hot-deck methodologies give real values with marginal distributions that are closer to the original ones.

In this case, where donors and recipients are random extractions of the same population, we have seen that it is possible to merge observed and imputed data to obtain more accurate estimates of prevalences. However, we would like to stress that this is not a general result, as it depends on various factors. First, the goodness of the imputation method must be considered. Also, calculating the variance of prevalences depends on the trade-off between the gain obtained in the within variability (due to the increased size) and the loss that occurs after adding the multiple imputations’s between variability.

## Conclusions

In this work we have shown that Data Fusion allows us to integrate two sources of information in order to better take advantage of the available data.

The imputation should always be multivariate (or sequential), to preserve the association between variables.

We generally use stochastic imputation to obtain simulated instances of reality and to preserve the variability of imputed values.

We have shown the advantages of using multiple imputation rather than single imputation, in order to deal with the uncertainty problem and provide better and more stable validation statistics.

We have proposed a methodology for choosing the most suitable imputation model for a given specific problem, based on a suite of validation statistics. Even if the imputation methodology is chosen because of the user’s concerns, the suite of validation statistics have empirically demonstrated their ability to indicate out the adequacy of an imputation technique for a specific problem.

Using fused data (original data plus the imputed data) it is possible to obtain more accurate estimates of the prevalences than by using just the observed data.

## Endnote

^1^ In general we will not have observed *Y*_1_. Consequently, we will be limited to approximating the discrepancy by performing the imputation on the donors. If we have the recipients’ information, *Y*_1_, we can compare $\,\hat {f}(X_{1},\hat {Y}_{1})$ with $\,\hat {f}(X_{1},Y_{1})$.

## Abbreviations

ACDe: Average correlation difference of the external coherence
ACDi: Average correlation difference of the internal coherence
ASD: Average Smirnov distance
ASLm: Average significance level for the mean
ASLs: Average significance level for the standard deviation
BMI: Body mass index
BP: Blood pressure
CVD: Cardiovascular diseases
DA: Data augmentation
DBP: Diastolic blood pressure
ESCA: Health survey of Catalonia
EXCA: Health exam data of Catalonia
MI: Multiple imputation
RndRes: Randomness of residuals
Ratio of SD: Ratio of the standard deviation prevalence of the observed data over the standard deviation prevalence of the imputed plus observed data
SQ-reg: Sequential regression
SBP: Systolic blood pressure
TAU: Prediction error statistic
WC: Weighted correlation
